# An Exploratory Study of Police Officers’ Perceptions of Health Risk, Work Stress, and Psychological Distress During the COVID-19 Outbreak in China

**DOI:** 10.3389/fpsyg.2021.632970

**Published:** 2021-03-19

**Authors:** Qiufeng Huang, Ali Ahmad Bodla, Chiyin Chen

**Affiliations:** ^1^School of Political Science and Public Administration, Huaqiao University, Quanzhou, China; ^2^School of Economics and Management, Tongji University, Shanghai, China; ^3^The Glorious Sun School of Business and Management, Donghua University, Shanghai, China

**Keywords:** police officers, health risk perception, work stress, psychological distress, COVID-19

## Abstract

**Background:**

How do the police officers perceive health risk, psychological distress, and work stress during the COVID-19 outbreak in China? This study explores the health risk perception, work stress, and psychological distress of police officers who worked at the front line to implement lockdown measures.

**Materials and Methods:**

We conducted a large-scale field survey (*N* = 5,611) with police officers sample in the northwestern part of China from February 29 to March 7, 2020. Independent-sample *T*-test and ANOVA were used to analyze whether there are differences in health risk perception, work stress, and psychological distress between different groups. The regression analysis was employed to figure out the factors that influence police officers’ psychological distress.

**Results:**

Results showed a gender difference in perceiving work stress among police officers. Also, police officers with chronic disease perceived higher health risks, more psychological distress, and higher work stress. Additionally, police officers above 45 years old significantly perceived higher health risks than young officers did. It also revealed that working hours contribute to police officers’ health risk perception, psychological distress, and work stress. Finally, our results highlight that age, working hours, chronic disease, health risk perception, and work stress significantly contribute to police officers’ psychological distress.

**Conclusion:**

Our research verifies that there is a gender difference in perceiving work stress among police officers. Police officers with ongoing medical issues and above 45 years old suffer more during the COVID-19 outbreak in China. Our research suggests that the government should pay more attention to their physical health and mental health. The heavy workload containing the COVID-19 extends police officers’ working hours, causing higher health risks, work stress, and psychological distress. This study contributes to the psychological distress literature and provides a way forward to other countries struggling to contain the COVID-19.

## Introduction

Since the outbreak of COVID-19, the front-line personnel, especially doctors, paramedical staff, and law enforcement individuals, have faced substantial health risks, psychological distress, and work stress (Kang et al., 2020; Lu et al., 2020). Due to the rapid spread of the COVID-19, the Chinese government took varying levels of restrictive measures (travel ban, 14-day quarantine, and lockdown), which increased police officers’ burden to enforce the restrictions (Graham-Harrison and Kuo, 2020; Wang D. et al., 2020). The government announced the cancelation of all Chinese Spring Festival celebrations and temporarily closed most public and private institutes. Furthermore, citizens should comply with home quarantine requirements until the pandemic’s effective containment (Lin et al., 2020; Tian et al., 2020).

As COVID-19 is a distinct clade from the beta coronaviruses related to human severe acute respiratory syndrome (SARS) and the Middle East respiratory syndrome (MERS) (World Health Organization, 2020), it spreads mostly through human-to-human contact (Chan et al., 2020; Wu et al., 2020). The front-line police officers went to the community to help quarantined residents, fought crimes during the epidemic, and conducted 24-h safety inspections of passing persons and vehicles at a checkpoint, which increased the possibility of contacting infected people. As of February 26, 2020, 400 police officers got infected in Wuhan, China (National Healty Commission of the People’s Republic of China, 2020). The heavy workload and life-threatening circumstances raise anxiety, psychological distress, and work stress in the front-line workforce (Ivana et al., 2017). The Ministry of Public Security of China issued a Handbook for Public Security Policemen to protect their physical and mental health at work during the COVID-19 outbreak (The Ministry of Public Security of the People’s Republic of China, 2020). However, research studies to date overlooked the mental health status of front-line police officers facing the COVID-19. Additionally, it is significant to understand how police officers perceive health risk, work stress, and psychological distress during the pandemic.

This study assesses the police officers’ perception of health risk, work stress, and psychological distress during the COVID-19 outbreak in China. Also, it explores whether different groups, based on their demographic characteristics, perceived health risk, work stress, and psychological distress differently or not. It examines factors that significantly influence police officers’ psychological distress. This study used large-scale survey data to assess the police officers’ perceptions of health risk, work stress, and psychological distress during the COVID-19 outbreak.

This study contributes to the extant literature in four ways. First, exploring police officers’ health risk perception, work stress, and psychological distress contributes to understanding police officers’ psychological states during a pandemic. Second, a better understanding of how various police personnel groups perceive the health risk, work stress, and psychological distress differently help design strategies to utilize police personnel during the pandemic effectively. Third, examining the effect of age, gender, work time, health status, health risk perception, and work stress on psychological distress facilitates to explain what factors influence the police officers’ psychological states significantly. Fourth, research findings can support target interventions to facilitate police officers’ psychological health and work outcomes.

## Materials and Methods

### Participants

Police offices of the Ningxia Hui Autonomous Region (Ningxia), a province in the northwestern part of China, participated in this study. Each Chinese province runs its police work within the state. The government of Ningxi hierarchically divides police officers into three levels, e.g., city, county, and township government. Each police officer level is responsible for all policing activities for its hierarchical state under the top-level leadership. Policing work includes traffic control, crime investigations, service to citizens, and managing law and order situation. During the COVID-19 crisis, police officers implemented local shutdowns, enforced home-quarantine policy, and encouraged social distancing. There are 150,000 permanent police officers from managerial and non-managerial positions in Ningxia province to serve a population of 6.946 million in five cities. Police departments can be divided into administrative and service departments depending on whether they directly contact the public. The number of officers in each department varies by the city, type of police work, and administrative level. Police officers are a part of China’s army, who have a strong spirit of complying with the superiors’ orders.

#### Procedures

Our study is part of a voluntary health investigation in a large-scale field study project. For data collection, the researcher first sent informed consent after explaining the research purpose to the local public security department leader. Data collection in this study began on February 29, 2020, 1 month into the COVID-19 emergency in China, and the total number of confirmed cases was 79,389. The cross-sectional survey was completed on March 7, 2020, and the total number of confirmed cases was 80,695 in China. After getting approval from the university ethics committee, we undertook a systematic random sample of 5,650 officers from approximately 16,000 officers. The sample used the employment roster. Every third officer on the employment roster participated in the study. We collected data *via* Wenjuanxing^1^ during the working hours, and the participants anonymously self-rated a questionnaire distributed over the internet. All subjects provided informed consent electronically before completing the questionnaire. A total of 5,650 questionnaires were collected, of which 5,611 were completed and returned, resulting in a response rate of 96%.

### Measures

#### Psychological Distress

We measured psychological distress using the six-item scale developed by Kessler et al. (2002); the items are present in Appendix A. Survey items were initially developed and validated in English, which has been used in annual government health surveys in the United States, Canada, and in the WHO World Mental Health Surveys (Kessler et al., 2002). The survey was translated into the Chinese language using a standard back-translation process (Brislin, 1986) and validated in China (Chan et al., 2000). The respondents rated their response using the five-point Likert scale ranging from 1 (strongly disagree) to 5 (strongly agree). The sample items are “loss of appetite within the last 2 weeks” and “sleep disorder within the last 2 weeks.” Factor analysis indicated that all the six items loaded on a single factor, and the lowest factor loading score was 0.78. The Cronbach’s alpha value was 0.90.

#### Health Risk Perception

In this study, health risk perception refers to an individual’s subjective judgment and evaluation of being infected with COVID-19. Participants rated their health risk perception of COVID-19 using two items adapted from the risk perception developed by Dai et al. (2020). The items included “I am very likely to be infected during the work” and “I feel quite dangerous of being infected during the work.” The participants responded on the five-point Likert scale ranging from 1 (strongly disagree) to 5 (strongly agree). Factor analysis indicated that two items loaded on a single factor, and the lowest factor loading score was 0.87. The Cronbach’s alpha value was 0.70.

#### Work Stress

Participants assessed the stress for doing their job during the COVID-19 outbreak using one question. The question is “Please indicate the level of work stress during the COVID-19 outbreak.” The participants responded on a five-point scale ranging from 1 (very low) to 5 (very high).

The participants also provided their essential demographic characteristics, such as age, sex, and location. Because COVID-19 is more dangerous for people with chronic disease (Chen N. et al., 2020; Wang C. et al., 2020), police officers with ongoing medical issues would suffer more negative health experiences at work. Participants were required to report whether they had any chronic disease to assess the self-perceived health status. The answer to this question was yes or no. We also asked the participants to report their daily working time during the past week.

### Data Analysis

The data were analyzed using the SPSS version18.0 program (IBM, Corp., Armonk, NY, United States). We report the descriptive statistics of the study variables in Table 1. Independent-sample *T* test and ANOVA were used to analyze whether there are differences in health risk perception, work stress, and psychological distress between different groups. Additionally, the regression analysis was employed to determine the factors that influence police officers’ psychological distress. Statistical significance was set at *p* < 0.05.

**TABLE 1:** Descriptions of the participants (*N* = 5,611).

Variables	Categories	Number	Percentage
Gender	Male	4,299	76.6%
	Female	1,312	23.4%
Chromic disease	Yes	1,481	26.4%
	No	4,130	73.6%
Age	Below 30 years	2,655	47.3%
	30–45 years	2,057	36.7%
	Above 45 years	897	16.0%
Work time	Below 8 h	1,910	34.0%
	8–11 h	1,473	26.3%
	12–14 h	1,496	26.7%
	Above 14 h	732	13.0%

*The age ranges from 18 to 60 (retirement age in law); the work time ranges from 5 to 23 h.*

## Results

### Demographics and Descriptive Statistics

Supplementary Table 1 presents the descriptive characteristics of the participants. Of the participants, 76.6% were male, and 26.4% had a chronic disease, while 73.6% were physically healthy. In terms of work time, 32.3% of participants worked below 8 h a day, 27% of participants worked 8–11 h per day, 27.4% of participants had worked 12–14 h per day, and 13.4% of the participants worked above 14 h per day. The average age was 34.14 years for all participants, 47.3% of participants were below 30 years old, 36.7% were 30–45 years old, and 16.0% were above 45 years old.

### *T*-Test and ANOVA of Different Groups

#### Comparison by Gender

We compared genders to examine whether male and female police officers perceive health risk, psychological distress, and work stress differently or not. We investigated the mean of health risk perception, work stress, and psychological distress between males and females. As shown in Table 2, there is no significant difference in the health risk perception (*T* = 0.96, *p* > 0.1) and psychological distress (*T* = 0.80, *p* > 0.1) between male and female groups. However, the work stress difference between males and females was significant (*T* = 2.29, *p* < 0.01). Specially, male police officers reported higher work stress (Mean = 2.95, SD = 1.21) than females did (Mean = 2.87, SD = 1.15).

**TABLE 2:** Comparison of the average level of health risk perception, work stress, and psychological distress between different genders.

Variables	Male (*N* = 4,299)	Female (*N* = 1,312)	*T*	*p* value
Health risk perception	2.48 ± 1.06	2.45 ± 1.03	0.955	0.34
Work stress	2.95 ± 1.21	2.87 ± 1.15	2.35*	0.019
Psychological distress	2.45 ± 1.01	2.42 ± 0.98	0.789	0.43

*p* value for two independent samples Mann–Whitney *U* test. *p < 0.05.

#### Comparison by Physical Health Status

We compared the difference in health risk perception and mental health between physically healthy police officers and police officers with chronic disease.

As the data show in Table 3, there is a significant difference in the health risk perception (*T* = 9.74, *p* < 0.01), work stress (*T* = 13.98, *p* < 0.01), and psychological distress (*T* = 21.77, *p* < 0.01) between the physically healthy police officers and those with chronic disease. Police officers with chronic disease perceived higher health risk (Mean = 2.70, SD = 1.07), more work stress (Mean = 3.30, SD = 1.17), and more psychological distress (Mean = 2.91, SD = 0.98) than physically healthy police officers did.

**TABLE 3:** Comparison of the average level of health risk perception, work stress, and psychological distress between different physical health status.

Variables (chronic disease)	Yes (*N* = 1481)	No (*N* = 4130)	*T*	*p* value
Health risk perception	2.70 ± 1.07	2.39 ± 1.04	−9.64***	<0.001
Work stress	3.30 ± 1.17	2.80 ± 1.19	−13.98***	<0.001
Psychological distress	2.91 ± 0.98	2.28 ± 0.96	−21.53***	<0.001

*p* values for two independent sample Mann–Whitney *U* test. ***p < 0.001.

#### Comparison by Age

The legal working age is from 18 to 60 years in China. We grouped participants by three categories: under 30 years old (age ≤ 30), 30–45 years old (30 < age ≤ 45), and above 45 years old (age > 45). We compared the average level of health risk perception, work stress, and psychological distress among the three groups. As shown in Table 4, there was a significant difference in health risk perception (*F* = 3.47, *p* < 0.01) among different age groups. *Post hoc* comparisons showed that police officers who were above 45 years old reported higher health risk perception (Mean = 2.56, SD = 1.01) than police officers below 30 years old (Mean = 2.45, SD = 1.07). Unfortunately, there was no significant differences in work stress (*F* = 0.18, *p* > 0.1) or psychological distress (*F* = 1.22, *p* > 0.1) among different age groups.

**TABLE 4:** One-way ANOVA of health risk perception, work stress, and psychological distress among different age groups of police officers.

Variables	Below 30 (*N* = 2,655)	30–45 (*N* = 2,057)	Above 45 (*N* = 897)	*F*	*p* value
Health risk perception	2.45 ± 1.07	2.47 ± 1.06	2.56 ± 1.01	3.47*	0.03
Work stress	2.93 ± 1.18	2.94 ± 1.21	2.92 ± 1.24	0.18	0.84
Psychological distress	2.43 ± 1.03	2.47 ± 0.99	2.47 ± 0.94	1.22	0.30

*p < 0.05.

#### Comparison by Work Time

Effectively implementing lockdown restrictions, 67.8% of participants worked longer than the normal days during the COIVD-19 outbreak. We group participants by work time: below 8 h/day (work hours ≤ 8), 8–11 h/day (8 < work hour ≤ 11), 12–14 h/day (11 < work hour ≤ 14), and above 14 h (work hour > 14). As indicated in Table 5, the ANOVA showed that the differences in health risk perception (*F* = 39.15, *p* < 0.01), work stress (*F* = 166.99, *p* < 0.01), and psychological distress (*F* = 153.22, *p* < 0.01) among different groups were significant. We ranked the health risk perception score, work stress score, and psychological distress score of each group as follows: above 14, 11–14, 8–11, and below 8 h. The result indicated that police officers who worked longer reported high health risk perception, work stress, and psychological distress.

**TABLE 5:** One-way ANOVA of health risk perception, work stress, and psychological distress among different work time groups.

Variables	Above 14 h (*N* = 732)	12–14 h (*N* = 1,496)	8–11 h (*N* = 1,473)	Below 8 h (*N* = 1,910)	*F*	*p* value
Health risk perception	2.75 ± 1.14	2.56 ± 1.08	2.48 ± 1.01	2.29 ± 1.00	39.15**	<0.001
Work stress	3.48 ± 1.23	3.15 ± 1.19	3.00 ± 1.10	2.50 ± 1.12	166.99**	<0.001
Psychological distress	2.93 ± 1.07	2.60 ± 1.00	2.50 ± 0.94	2.11 ± 0.90	153.22**	<0.001

**p < 0.01.

### OLS Regression Analysis of Factors Influencing Police Officers’ Psychological Distress

Table 6 presents the descriptive statistic and correlations of our study variables. The mean values of health risk perception and the participants’ work stress were 2.47 (SD = 1.06) and 2.93 (SD = 1.20). Among the variables, chronic disease (*r* = 0.279, *p* < 0.01), work time (*r* = 0.237, *p* < 0.01), health risk perception (*r* = 0.534, *p* < 0.01), and work stress (*r* = 0.593, *p* < 0.01) had moderate statistically positive correlations with psychological distress, whereas gender (*r* = −0.01, *n.s.*) and age (*r* = 0.018, *n.s.*) had non-significant correlations.

**TABLE 6:** Descriptive statistics and correlations.

	Variables	Mean	SD	1	2	3	4	5	6
1	Gender (Male = 0, Female = 1)	0.23	0.42	1					
2	Age	34.14	9.40	−0.037**	1				
3	Chronic disease (No = 0, Yes = 1)	0.26	0.44	−0.067**	0.410**	1			
4	Work time	10.73	3.85	−0.248**	−0.039**	0.087**	1		
5	Health risk perception	2.47	1.06	–0.013	0.029*	0.129**	0.124**	1	
6	Work stress	2.93	1.20	−0.031*	–0.006	0.184**	0.255**	0.425**	1
7	Psychological distress	2.45	1.00	–0.01	0.018	0.279**	0.237**	0.534**	0.593**

*p < 0.05 and **p < 0.01.

Collinearity exists when variables overlap invariance, such as a correlation between two variables is higher than 0.70, which could affect regression results. The results in Table 6 showed that the highest correlation is 0.593, and the results in Table 7 showed that the highest variance inflation factor (VIF) score is 1.317. Collinearity is not a concern in this study.

**TABLE 7:** OLS regression analysis of factors influencing psychological distress.

	Model 1				Model 2				
Variables	*B*	SE	*T*	*p* value	*B*	SE	*T*	*p* value	VIF
Gender (Male = 0, Female = 1)	0.146***	0.03	4.80	0.001	0.088***	0.024	3.72	0.001	1.071
Age	−0.1***	0.001	–6.98	0.001	−0.006***	0.001	–5.62	0.001	1.218
Chronic disease (No = 0, Yes = 1)	0.69***	0.03	22.05	0.001	0.42***	0.025	16.89	0.001	1.263
Work time	0.06***	0.003	17.21	0.001	0.022***	0.003	8.30	0.001	1.147
Health risk perception					0.315***	0.10	31.27	0.001	1.226
Work stress					0.33***	0.009	35.92	0.001	1.317
Model *F*	217.77				869.02				
*R* square	0.135				0.482				
*R* square change					0.348				

*B*, unstandardized beta; VIF, variance inflation factor; *N* = 5,611. ***p < 0.001.

Table 6 presents ordinary least squares (OLS) regression analysis results of factors influencing police officers’ psychological distress during the COIVD-19 outbreak. Model 2 estimated psychological distress as the dependent variable and gender, age, chronic disease, work time, health risk perception, and work stress as independent variables. The *R* square value in Model 2 was 0.482, which means all the independent variables explained 48.2% of the observed variance in police officers’ psychological distress measure.

The results indicate that the police officers with chronic disease, in contrast to physically healthy police officers, experienced higher psychological distress (*B* = 0.69, *p* < 0.001). Work time had significant positive effects on psychological distress (*B* = 0.06, *p* < 0.001), indicating that police officers with longer work hours generally have more psychological distress. As shown in Model 2, health risk perception (*B* = 0.315, *p* < 0.001) and work stress (*B* = 0.009, *p* < 0.001) had significantly positive effects on psychological distress, indicating that those police officers with increasing health risk perception and work stress perceive higher psychological distress.

## Discussion

This study explored the police officers’ perceptions of health risk, work stress, and psychological distress during the COVID-19 outbreak in China. It also examined how police officers’ perceptions vary depending on their health status, work time, and demographic characteristics. This study revealed interesting findings based on a large data set (*N* = 5,611) of specific front-line personnel, police officers during the COVID-19 outbreak in China. Although policemen and policewomen do not differ in perceived health risk and psychological distress, male officers perceive significantly higher work stress than female officers. Our research verifies a gender difference in perceiving work stress among police officers (Kim et al., 2016; Violanti et al., 2016). It might be because more male police officers work in the field and face extreme circumstances to implement restrictions. Therefore, this study contributes to the idea that male police officers perceive high work stress because of their work structure and nature (Chan et al., 2000; Lucas et al., 2012). The extant research finds that medical staff perceives greater fear, anxiety, and depression than administrative staff because the medical staff’s work structure and nature are different from those of administrative staff (Lu et al., 2020).

Furthermore, we found that police officers with chronic disease perceive higher health risks, psychological distress, and work stress. Initial findings of COVID-19 show that individuals with poor health conditions are more prone to the virus (Chen S. M. et al., 2020). Prior research mentioned that front-line employees with weak medical history were more likely to catch the virus (National Health Commission of the People’s Republic of China, 2020). Therefore, we contribute to the literature by explaining that police officers with chronic medical history are more concerned about the virus.

Additionally, this study analyzed whether police officers of different age groups perceive different levels of health risk, work stress, and psychological distress or not. Our finding revealed that police officers above 45 years old significantly perceive higher health risks than younger people. Our finding supports the evidence that old age individuals are usually more concerned and prone to health disease (Wong, 2014). Thus, to some extent, it complements our previous finding that police officers with chronic disease perceive higher health risk and psychological distress.

We also found those police officers who work longer hours perceive higher health risks, work stress, and psychological distress compared to those who work shorter hours. Prior research endorses our findings that the front-line doctors, paramedical staff, and police officers worked hard to contain the COVID-19 virus in China (Kang et al., 2020; Lu et al., 2020). Due to a shortage of administrative and medical staff in Wuhan, front-line personnel were relocated from other provinces to ease the existing staff workload (National Health Commission of the People’s Republic of China, 2020). Longer working hours negatively influence police officers even though the Chinese government supports the front line personnel to cope with anxiety, work stress, and psychological distress (Tian et al., 2020).

Finally, this study found that police offers that work longer hours, have a chronic disease, and with older age significantly feel psychological distress. Furthermore, police officers’ perceptions of health risk and work stress positively influence psychological distress. This study contributes to the psychology literature and provides a way forward to other countries struggling to contain the COVID-19. Our findings will help crisis management managers to plan and develop strategies to contain a crisis effectively. During a health crisis or pandemic, police officers work intensively at the front line. Because our finding suggests that police officers with chronic disease perceive higher health risk, work stress, and psychological distress, we suggest that work arrangements should be made according to police officers’ health conditions.

We are reasonably confident about the generalizability of our research finding because of several reasons. First, we used a large sample size (5,611) of police officers to analyze health risk, psychological distress, and work stress during the COVID-19 outbreak in China. Second, rigorous research methodology and reliability confirmation of measurements support our results’ validity. It encouraged us to draw evidence-based inferences from the research results. However, our study has certain limitations worthy of future research. First, in this study, the participants were from one province in China. Therefore, it should be treated with caution when generalizing the results to the police officer groups outside China. Future research should use a sample of different front-line personnel from other countries to validate the research findings. Second, the research relied on cross-section data, and the design limits the discussion on the changes in health risk perception and mental health of police officers during the epidemic. Future research could employ a longitudinal research design to explore research causality. Third, compared with objective data, self-reported data may contain social desirability bias. Future research should use two or more data sources to avoid social desirability and common method bias issues. Finally, although we confirmed the constructs’ validity and reliability, several variables used in our study are adapted from the English version.

## Conclusion

This study revealed that front-line personnel, especially police officers, perceived health risk, work stress, and psychological distress during the COVID-19 outbreak in China. Furthermore, it suggests that old age, long working hours, and a chronic disease significantly contribute to police officers’ psychological distress. A better understanding of the police officers’ concerns during the outbreak can help develop management practices to improve individuals’ psychological health. This study opens several avenues for future research to manage front-line personnel during a pandemic.

## Data Availability Statement

The original contributions presented in the study are included in the article/Supplementary Material, further inquiries can be directed to the corresponding author/s.

## Ethics Statement

The studies involving human participants were reviewed and approved by the Ethics Committee at Huaqiao University (#20200229). The participants provided their written informed consent to participate in the study.

## Author Contributions

QH conceptualized the study, carried out the initial analyses and interpreted the data, review and revised the manuscript, and approved the final manuscript as submitted. AB contributed to draft the initial manuscript and approved the final manuscript as submitted. CC contributed to the writing and editing. All authors contributed to the article and approved the submitted version.

## Conflict of Interest

The authors declare that the research was conducted in the absence of any commercial or financial relationships that could be construed as a potential conflict of interest.

## Footnotes

1 www.wjx.cn

## Appendix A

1. How often did you feel so depressed that nothing could cheer you up?
2. How often did you feel restless or fidgety?
3. How often did you feel that everything was an effort?
4. How often did you feel nervous?
5. Loss of appetite within the last 2 weeks.
6. Sleep disorder within the last 2 weeks.

## References

[B1] Brislin R. W. (1986). “The wording and translation of research instruments,” in *Field Methods in cross-cultural research*, eds Lonner W. J. Berry J. W. (Beverly Hills, CA: Sage), 137–164.

[B2] Chan J. F. Yuan S. Kok K. H. To K. W. Chu H. Yang J. (2020). A familial cluster of pneumonia associated with the 2019 novel coronavirus indicating person-to-person transmission: a study of a family cluster. *Lancet* 395 514–523. 10.1016/S0140-6736(20)30154-9 31986261 PMC7159286

[B3] Chan K. B. Lai G. Ko Y. C. Boey K. W. (2000). Work stress among six professional groups: the Singapore experience. *Soc. Sci. Med.* 50 1415–1432. 10.1016/S0277-9536(99)00397-4 10741577

[B4] Chen N. Zhou M. Dang X. Gong F. Han Y. Qiu Y. (2020). Epidemiological and clinical characteristics of 99 cases of 2019 novel coronavirus pneumonia in Wuhan, China: a descriptive study. *Lancet* 395 507–513. 10.1016/S0140-6736(20)30211-7 32007143 PMC7135076

[B5] Chen S. M. Zhang Z. J. Yang J. T. (2020). Fangcang shelther hospitals:a novel concept for responding to public health emergencies. *Lacent* 395 1305–1314. 10.1016/S0140-6736(20)30744-3 PMC7270591 32247320

[B6] Dai Y. J. Hao Y. H. Wu Q. H. Wang X. Y. Wang X. F. Chen C. Y. (2020). Estabblishment and evaluation on reliability and validity of public risk percetpion scale for public health emergencies. *Chin. J. Public Health* 36 227–231.

[B7] Graham-Harrison E. Kuo L. (2020). *China’s Coronavirus Lockdown Strategy: Brutal But Effective.* Available online at: https://www.theguardian.com/world/2020/mar/19/chinas- coronavirus-lockdown-strategy-brutal-but-effective (accessed June, 2020).

[B8] Ivana M. I Arandjelović M. Ž Jovanović J. M. Nešić M. M. (2017). Relationships of work-related psychosocial risks, stress, individual factors and burnout – questionnaire survey among emergency physicians and nurses. *Medycyna. Pracy.* 68 167–178. 10.13075/mp.5893.00516 28345677

[B9] Kang L. Ma S. Chen M. Yang J. Wang Y. Li R. T. (2020). Impact on mental healthy and perceptions of psychological care among medical and nursing staff in Wuhan during the 2019 novel coronavirus disease outbreak: a cross-section study. *Brain Behav. Immun.* [Epub ahead of print]. 10.1016/j.bbi.2020.03.028 PMC7118532 32240764

[B10] Kessler R. C. Andrews G. Golpe L. J. Hiripi E. Mroczek D. K. Normand S. T. (2002). Short screening scales to monitor population prevalences and trends in non-specific psychological distress. *Psycho. Med.* 32 959–976. 10.1017/S0033291702006074 12214795

[B11] Kim J. L. Wells W. Vardalis J. J. Joohnson S. K. Lim H. (2016). Gender difference in occupational stress: a study of the south korean national police agency. *Int. J. Law Crime Justice* 44 163–182. 10.1016/j.ijlcj.2015.09.001

[B12] Lin Q. Y. Zhao S. Gao D. Z. Lou Y. Yang S. Musa S. S. (2020). A conceptual model for the coronavirus disease 2019 (COVID-19) outbreak in Wuhan, China with individual reaction and government action. *Int. J. Infect. Dis.* 93 211–216. 10.1016/j.ijid.2020.02.058 32145465 PMC7102659

[B13] Lu W. Wang H. Lin Y. Li L. (2020). Psychological status of medical workforce during the COVID-19 pandemic: a cross-sectional study. *Psychiatry Res.* 288:112936. 10.1016/j.psychres.2020.112936 32276196 PMC7195354

[B14] Lucas T. Weidner N. Janisse J. (2012). When does work stress come from? A generalizability analysis of stress in police officers. *Psychol. Healty* 27 1426–1447. 10.1080/08870446.2012.687738 22612444

[B15] National Health Commission of the People’s Republic of China (2020). *Occupational Injury Insurance Supports Occupational Injury “Protection Umbrella” for Prevention and Rescue Personnel in the Fight Against New Coronavirus Pneumonia, 2020b.* Available online at: http://www.nhc.gov.cn/xcs/fkdt/202001/0ffbb879ac1b4466b01de8b40a1372fc.shtml (accessed April 26, 2020).

[B16] National Healty Commission of the People’s Republic of China (2020). *Reporting outbreaks.* Available online at: http://www.nhc.gov.cn/xcs/yqtb/list_gzbd.shtml (accessed April 5, 2020).

[B17] The Ministry of Public Security of the People’s Republic of China (2020). Available online at: https://www.mps.gov.cn/n2255079/n6865805/n6888406/n6888452/c6914329/content.html (accessed February 20, 2020).

[B18] Tian F. Li H. Tian S. Yang J. Shao J. Tian C. (2020). Psychological symptoms of ordinary Chinese citizens based on SCL-90 during the level I emergency response to COVID-19 public health emergency of international concern. *Psychiatry Res.* 288:112992. 10.1016/j.psychres.2020.112992 32302816 PMC7151383

[B19] Violanti J. M. Fekedulegn D. Hartley T. A. Charles L. E. Andrew M. E. Ma C. C. (2016). Highly reated and most frequent stressors among police officers: gender difference. *Am. J. Crim. Just.* 41 645–662. 10.1007/s12103-016-9342-x 28260848 PMC5330309

[B20] Wang C. Horby P. W. Hayden F. G. Gao G. F. (2020). A novel conronavirus outbreak of global health concerin. *Lancet* 395 470–473. 10.1016/S0140-6736(20)30185-9 31986257 PMC7135038

[B21] Wang D. Hu B. Hu C. Zhu F. Liu X. Zhang J. (2020). Clinical characteristics of 138 hospitalized patients with 2019 novel coronavirus–infected pneumonia in Wuhan, China. *JAMA* 323 1061–1069. 10.1001/jama.2020.1585 32031570 PMC7042881

[B22] Wong T. Y. (2014). Global prevalence of age-related macular degeneration and disease burden projection for 2020 and 2040: a systematic review and meta-analysis. *Lancent Glob. Healty* 2 e106–e116. 10.1016/S2214-109X(13)70145-1 25104651

[B23] World Health Organization (2020). *Statement on the second meeting of the International Healty Regulations (2005) Emergency Committee regarding the outbreak of novel coronavirus (2019-nCoV).* Available online at: https://www.who.int/news-room/detail/30-01-2020-statement-on-the-second-meeting-of-the-international-health-regulations-(2005)-emergency-com-mittee-regarding-the-outbreak-of-novel-coronavirus-(2019-ncov) (assessed February, 15, 2020).

[B24] Wu J. T. Leung K. Leung G. M. (2020). Nowcasting and forecasting the potential domestic and international spread of the 2019-nCoV outbreak originating in Wuhan, China: a modelling study. *Lancet* 395 689–697. 10.1016/s0140-6736(20)30260-9 32014114 PMC7159271

